# Personality Traits Predict Social Network Size in Older Adults

**DOI:** 10.1177/01461672221078664

**Published:** 2022-04-08

**Authors:** Jasmine Rollings, Jérôme Micheletta, Darren Van Laar, Bridget M. Waller

**Affiliations:** 1 University of Portsmouth, UK; 2 Nottingham Trent University, UK

**Keywords:** social networks, social groups, older adults, extraversion, agreeableness, communication

## Abstract

Humans live in unusually large groups, where relationships are thought to be maintained through complex socio-communicative abilities. The size and quality of social networks are associated with health and well-being outcomes throughout life. However, how some individuals manage to form larger social networks is not well understood. If socio-communicative traits evolved to form and maintain relationships, personality traits should be associated with variation in network size. Here, using the English Longitudinal Study of Ageing (ELSA), we investigate the impact of extraversion, agreeableness, and verbal communication on network size (*N* = 5,202) and network size change over time (*N* = 1,511) in later life for kin and friend networks. Higher levels of extraversion and agreeableness were associated with greater social network sizes but did not predict network size change over 14 years. The findings are discussed considering the evolutionary hypothesis that communicative and affiliative traits may have evolved to support the maintenance of social networks.

## Introduction

An abundance of research has linked larger social networks to positive physical and mental health outcomes. Larger social networks can be protective against depression (Santini et al., 2015) and are associated with increased happiness (Chan & Lee, 2006), better life satisfaction (Huxhold et al., 2013), reduced sleep disturbance and fatigue (Cho et al., 2019), as well as reduced mortality risk (Holt-Lunstad et al., 2015). Meta-analytic findings have also shown that strong social relationships (based on structural qualities such as network size as well as quality) are associated with decreased mortality risk, over and above more proximal health indicators, such as body mass index (BMI) smoking, alcohol consumption, and physical activity (Holt-Lunstad et al., 2010). Larger social networks are likely associated with these various positive outcomes because they allow the individual to access social resources in the form of instrumental and emotional support (Van Tilburg, 1995). However, despite the crucial role of social networks in life outcomes, the factors that are predictive of individual differences in social network sizes are not well understood. The fundamental psychological processes underpinning social network formation and maintenance need to be understood if we are to understand how people engage with these processes at an individual level.

### Theoretical Framework

Individual differences in human social network size are well documented (e.g., Dunbar & Spoors, 1995; Pollet et al., 2011). The social brain hypothesis (Dunbar, 1998; Freeberg et al., 2019) offers a framework within which to understand these individual differences in social network formation. Proponents argue that human and non-human primates have developed large brains and superior social abilities to handle the demands of living in complex social groups. Species in large social groups encounter more demanding social interactions, such as the requirement to deceive, form coalitions and maintain group cohesion (Dunbar & Shultz, 2007); these species have larger brain sizes than species operating in smaller, simpler social systems. Neuroimaging studies indicate that the social brain hypothesis is not only applicable across species, but also within humans. For example, the amygdala is implicated in social communication skills, and amygdala volume has been found to positively correlate with social network size and complexity (Bickart et al., 2011). Furthermore, the structure of focal regions of the human brain—that have been identified as important for social living—are similarly associated with the number of online social contacts (Kanai et al., 2012). Thus, individual differences in brain structure associated with socio-communicative skills and traits are predictive of the number of network members someone has in their social network. However, a direct relationship between socio-communicative traits and social network size has not yet been clearly demonstrated.

Social networks are characterized as being made up of group layers corresponding to different levels of intimacy (Hill & Dunbar, 2003). The innermost subgroup is the support clique—People the focal individual feels the closest to and could turn to for advice or support. The next layer is the sympathy group, made up of friends and kin, sometimes defined as those whose death the individual would find upsetting (Buys & Larson, 1979). Finally, the outer layer of one’s social group: This group could include workplace acquaintances and neighbors (Hill & Dunbar, 2003). The average number of individuals in an individual’s total social network is approximately 125 (Hill & Dunbar, 2003). This corresponds closely to the estimated maximum number of network members of approximately 150, based on the relationship between group size and neocortex size in primates (Dunbar, 1993; Roberts et al., 2009). The size of each layer has been reported to lie between 3 and 5 for the support clique, 12 and 20 for the sympathy group, and 30 and 50 for outer layer network size (Zhou et al., 2005). It has been argued that this linear relationship between closeness and number of ties at each layer is a product of the costs of maintaining closer relationships (Sutcliffe et al., 2012). However, there is wide variation in the reported range of network sizes. This could be partly due to methodological differences between studies, but also due to the individual differences within and between the populations studied (Dunbar & Spoors, 1995; Hill & Dunbar, 2003).

Social networks are also characterized by changes over time, as network ties are gained and lost; however, the research concerning the relationship between social network size and aging are inconsistent. Some research indicates that network size depletes in later life, with a lifespan trajectory of network size gain in adolescence and young adulthood, reaching a plateau in one’s early 30s followed by a steady decline into later life (Wrzus et al., 2013). Others have suggested that total network size remains stable throughout later life (Van Tilburg, 1998), or contradictorily, that network sizes may be more likely to experience a period of growth in later life (Cornwell et al., 2014). Socioemotional selectivity theory (SST) suggests that as people age, and their perception of time horizons become more limited, people prioritize close relationships and form smaller, more meaningful social networks than their younger counterparts (Carstensen, 2021). This theory is likely to be more applicable to adults in late later-life, when time is perceived to be more finite, rather than younger older adults, so it would be expected that the older sample members would have smaller social network groups. In summary, previous findings suggest that the relationship between social network size and age may not follow a linear trajectory; particularly when studying a group that spans multiple stages of later late.

### Individual Differences and Social Network Size

Inter-individual variation in both total network size and at the different hierarchical layers, could be due, in part, to individual differences in socio-communicative characteristics. Research examining the link between communicative characteristics and social networks has focused almost exclusively on atypical populations, however. For example, individuals suffering from aphasia following a stroke were more likely to have smaller network sizes and communicate with fewer friends than those who did not develop aphasia (Davidson et al., 2008). Similarly, survivors of laryngeal cancer with poorer communication had a smaller social network (Blood et al., 1994). However, it can be difficult to isolate the impact communication can have on network size when there is comorbidity with physical disability. A new approach tackled this issue by controlling for health conditions and found that communication difficulty remained associated with smaller network sizes (Palmer et al., 2016). The latter study supports the hypothesis that communicative ability is an independent predictor of network size regardless of physical disability. As communication difficulty is more likely to be more prevalent as people age (Yorkston et al., 2010), it is important to investigate the impact communicative traits can have on older adults’ social networks.

There is evidence that aspects of personality and individual differences may also be predictive of network size, but the findings are inconsistent across age groups. In a study of young adults who were new university attendees, extraversion, agreeableness, and conscientiousness predicted the number of peer relationships and social support at frequent follow-ups (Asendorpf & Wilpers, 1998). Correspondingly, some studies found that those who were more extroverted had larger social network sizes at all levels of their network, including the support group, the sympathy group, and outer layers (Pollet et al., 2011). In contrast, others have found that extraversion is important at only some network layers—such as the support clique (Molho et al., 2016). However, some findings suggest that the positive relationship between extraversion and network size is not found once age is taken into account, perhaps due to a decline in extraversion as people age (McCrae & Terracciano, 2005; Roberts et al., 2008). Thus, there is a need to further examine the relationship between personality and social network size specifically in older people. Contrary to the findings with younger people, the link between extraversion and social network size in older adults is not as clearly defined or consistent. A study with older adults in the Netherlands found that the big five personality factors, including extraversion and agreeableness, were unrelated to network size in depressed and non-depressed participants once confounds such as age, relationship status, and health were controlled for (Schutter et al., 2019). Similarly, a study using a longitudinal nationally representative sample of older adults in America found that while extraversion was reliably related to strength of social ties, the relationship between extraversion and social network size was weak (Iveniuk, 2019).

### The Present Study

The present study reports the results of two secondary analyses of the English Longitudinal Study of Ageing (ELSA). ELSA is a nationally representative cohort survey study of adults over the age of 50 years, collected in England from 2002 until present. We present cross-sectional single-wave and first–last change analyses to address whether socio-communicative traits predict social network size in older adults and investigate whether these traits are important for network size change in later life. Of the big five personality factors, extraversion and agreeableness are most applicable for research concerning interpersonal relationships: Extraverted individuals are outgoing, enthusiastic, and inclined to be sociable with others, and agreeable individuals are warm, kind, and sympathetic (McCrae & John, 1992). Therefore, as in previous studies (Jensen-Campbell et al., 2002; Tov et al., 2016), this study will focus on the personality traits extraversion and agreeableness. As previous research has highlighted the importance of communicative ability, we will also utilize ELSA data related to verbal communication. It is hypothesized that the socio-communicative traits—extraversion, agreeableness, and verbal communication—will be positively associated with network size in the cross-sectional analysis. Furthermore, it is expected that the socio-communicative traits found to be important to network size in the cross-sectional analysis, will be predictive of network size change over a 14-year period.

Some previous research has identified a need to separate out the kin and friendship network size, with the former being more likely to remain stable throughout the lifespan and the latter more volatile (Roberts & Dunbar, 2011). Likewise, research has found that while friendship network size decreases with age, family networks do not suffer the effects of age (Wrzus et al., 2013). This could in part be due to the costly nature of friendship relationships, which has been shown to be more vulnerable to decay when there is a lack of regular maintenance through shared activities and frequent contact (Roberts & Dunbar, 2011). Unlike most kin relationships, friendships undergo a formation process, which can vary across friendships, but usually involves an initial attraction to a potential friend followed by the sharing of thoughts, feelings, and experiences (Adams & Blieszner, 1994). This process relies upon successful communication during interactions, which could be aided by socio-communicative traits. Therefore, an exploratory analysis was conducted to separate the kin and the friendship relationships, to explore the impact socio-communicative traits have on these different network types.

## Methods

### Participants

The English Longitudinal Study of Ageing (ELSA) is a population-based longitudinal panel study of adults aged 50 years and above. ELSA data are collected from individuals bi-annually (Steptoe et al., 2013); at the time of writing, eight waves (data collection periods) were available. Wave 1 data were collected in 2002/2003 with an original sample of 12,099 participants. Sample size at each wave fluctuates as original participants leave the sample and replenishment participants join. The ELSA data cannot be made available in conjunction with this article due to copyright; however, the data are freely available to download from the U.K. data service (Oldfield et al., 2020) (see supplementary material for more information).

For the cross-sectional single-wave analysis, the sample included core members from Wave 5 of ELSA, the wave in which trait data were collected. Participants under the age of 50 (*n* = 355), who did not attend a full interview in person (*n* =567), and who did not provide responses to all the analysis variables (*n* = 1847) were excluded. This resulted in a sample size of 7,505 (55.7% female). Cross-sectional survey weights are only available in the ELSA for core members; therefore, other member types (such as core partners and younger partners) were excluded (*n* = 635). There was a high intra-class correlation between individuals within households (household members had similar network sizes), and so we randomly excluded one person per household in households that had two core members (*n* = 1,668). The final sample size for the cross-sectional analysis was 5,202 (57.8% female). Sample descriptives are provided in Table 1.

As the first–last change analysis investigated network size change from Waves 1 to 8, this sample included participants who were present in Waves 1 (baseline) and 8 (end). Participants also had to have been present in Wave 5 (when personality and verbal communication data were collected). This sample was extracted based on the cross-sectional analysis sample; therefore, those under 50, those who did not complete the full interview in person, and those who did not provide a response to the outcome/predictor variables had already been excluded. Of this clean data set, 4,524 participants were in either Wave 1 or 8; when this was filtered to only include those who were present in both waves, the final sample size was 1,511 (56.3% female). We report all manipulations, measures, and exclusions in these studies.

**Table 1.** Sample Characteristics Per Analysis.

	Analysis	
Variable	Cross-sectional analysis (*N* = 5,202)	Change analysis (*N* = 1,511)
Gender		
*Male*	2194 (42.2%)	660 (43.7%)
*Female*	3008 (57.8%)	851 (56.3%)
Age	66.8 (8.62) 65 [52, 89]	
Social network size	7.31 (4.98) 6 [0–79]	
Friend network size	3.72 (3.28) 3 [0–40]	
Family network size	4.15 (3.12) 4 [0–59]	
Age at baseline		59.4 (6.43) 58 [50–76]
Social network size change		0.07 (5.34) 0 [−62 to 36]
Friend network size change		0.3 (4.00) 0 [−68 to 27]
Family network size change		−0.02 (3.21) 0 [−25 to 30]
Extraversion	5.95 (1.47) 6 [2–8]	6.06 (1.41) 6 [2–8]
Agreeableness	7.05 (1.08) 7 [2–8]	7.06 (1.10) 7 [2–8]
Verbal Communication	3.07 (0.87) 3 [1–4]	3.07 (0.87) 3 [1–4]

*Note.* Count (percentage) presented for gender only. Mean (*SD*), median [range] presented for all other variables.

### Measures

#### Social network size, friend network size, and family network size

As in Rafnsson et al. (2015), we calculated social network size from the sum of three questions in ELSA, which ask the number of children, family members (other than spouse or children), and friends participants “felt close to.” The framing of these questions attributes intimacy with group members as participants are asked to report the number of children, family, and friends they “feel close to.” Therefore, social network size in this study is likely representative of the two innermost levels of an individual’s social network—the support clique and the sympathy group—based on the descriptions in the literature of these two layers (Hill & Dunbar, 2003).

Friend network size was the response to the question “how many friends do you feel close to?” to which participants provided a numerical response. Family network size was the sum of the numerical responses to the questions “how many of your children do you feel close to?” and “how many family members (other than your spouse or children) do you feel close to?”

#### Extraversion and agreeableness

Items included in ELSA were chosen based on similarity to the 10-item personality inventory (TIPI) (Gosling et al., 2003). In Wave 5 of ELSA, participants were asked to indicate how well particular personality traits describe them, indicating “A lot,” “Some,” “A little,” “Not at all,” giving a score of 1 to 4. This was reverse scored in the analysis so that four represented the highest score. For extraversion, the responses to the following traits were summed: “outgoing” and “lively.” For agreeableness, the scores for “warm” and “sympathetic” were summed. The sum of these variables resulted in scores of 2 to 8 for both extraversion and agreeableness. Internal consistency for these measures was acceptable (extraversion: Cronbach’s α = .70; agreeableness: α = .62). These values are similar, greater even, to those found with the TIPI (α = .68, α = .40) (Gosling et al., 2003).

#### Verbal communication

Verbal communication was based on responses to one item—how talkative the individual reported themselves to be. Based on the responses “Not at all, “A little,” “Some,” “A lot,” the verbal communication variable had a score from 1 to 4, with 1 being the least and 4 being the most talkative. This variable, as with the personality trait data, was only collected in Wave 5 of ELSA, therefore the individuals’ score was copied across waves for the change analysis. In this study “talkativeness” has been used as a proxy for verbal communication, to the authors knowledge this variable of the ELSA has not been used in this way before. Therefore, an additional online study with adults aged 50+ (*n* = 101) was performed to assess the measure’s concurrent validity with previously validated measures of communicative competence and preference (see supplementary material for full details). The results of the online study indicated that trait verbal communication was associated with self-assessed communicative competence, as assessed by the Interpersonal Communication Competence Scale (ICCS-SF: Rubin & Martin, 1994), and perceived communicative competence, as assessed by the Self-Perceived Communication Competence Scale (SPCC: McCroskey & McCroskey, 1988), though there was no clear relationship between verbal communication and communication preference, as assessed by the Willingness to Communicate scale (WTC: McCroskey & Richmond, 1987).

#### Covariates

Age, gender, relationship status, and general health were also included in the models. Socioeconomic status was included in the cross-sectional analysis only, due to inconsistencies in socioeconomic data types across waves. Gender was coded as 1 for male and 2 for female. Relationship status was based on whether an individual was single or cohabiting/married (coded as 2 and 1, respectively). General health could have a score of 1 to 5, representing answers “poor,” “fair,” “good,” “very good,” “excellent”, given in response to the question: “How is your health in general?” Employment data were collected in ELSA interviews; participants were scored from 1 to 8 (“Higher managerial and professional occupations” to “Never worked or long-term unemployment”). To ease interpretation of the regression outputs, this was reverse scored in the analysis so that a higher score reflected higher managerial and professional occupations. In the quadratic model, baseline age was transformed by the addition of a second-order polynomial—a quadratic term. In the ELSA data set, individuals aged 90+ were classed as 90 years old. ELSA research administrators apply this transformation to maintain anonymity, due to the small number of participants in this age range.

#### Survey weights

Cross-sectional survey weights for Wave 5 are available as part of the ELSA data set. These survey weights ensure representativeness of the sample, with respect to the participants propensity to respond and age according to the national population.

#### Social network size, friend network size, and family network size change

Network size change was calculated by assessing the difference between network size at Wave 1 (2002/2003) and Wave 8 (2016/2017). A positive number indicates an increased network size, while a negative number indicates a decreased network size over the 14-year period. The same procedure was carried out for social, friend, and family network size. For the binomial regression, each change score was converted into 0 or 1, 0 represented a decrease in network size and 1 represented either a stable network size (no change) or an increased network size. This grouping was chosen to assess whether socio-communicative traits would be protective against network size decline.

#### Baseline/end of study scores (change analysis only)

Baseline age was taken from Wave 1; final relationship status and health status were taken from Wave 8.

### Data Analysis

Data cleaning, preparation, and analysis were performed in R programming software (R Core Team, 2018) using version 4.1.0. Supplementary R scripts are available in the following repository: https://osf.io/2ua95/.

The cross-sectional analysis utilized linear regression modeling, while the change analysis utilized binomial logistic regression—R package “lme4,” version 1.1-27.1 (Bates et al., 2015). Survey weights were applied to the linear model using the R package “survey” (Lumlet, 2004). Models were compared using the “ANOVA” function in the R package “car” (Fox & Weisberg, 2011), which tests for a significant improvement in model fit using chi-square test of difference. Models were also compared based on *R*^2^ values and Akaike information criterion (AIC) values. VIF calculations indicated a lack of multicollinearity if all VIF values were below 2.5 (excepting quadratic terms), which is deemed a conservative acceptable level (Johnston et al., 2018). Assumptions checks are available to view in the supplementary materials. We used the inbuilt squaring function (^2) within R statistical software package “lme4” for adding higher-order polynomials. This allows the relationship between the outcome variable and the predictor to have a curvilinear relationship in the model. In the present study, we have utilized Cohen’s *f*^2^ to measure local effect sizes, which is calculated using the following formula (Selya et al., 2012):

$$f^{2}=\frac{R_{AB}^{2}-R_{A}^{2}}{1-R_{AB}^{2}}.$$

#### Single-wave analysis

All variables were input as fixed effects to predict social network size, friend network size, and family network size. In the case of all three analyses, the outcome variables (the network sizes) were log transformed to achieve normal distribution of model residuals, a constant of 1 was added to each network size to avoid exclusion of 0 responses.

##### Social network size

A null model (Model 1.1) formed of only the control variables was compared against a full model (Model 1.2) with the addition of the socio-communicative traits—extraversion, agreeableness, and verbal communication. The full model was also compared with a quadratic model (Model 1.3) which also considered the non-linear relationship between network size and age, which was indicated in visual exploration of the raw variables (see supplementary material). Additional models were also computed to assess the impact of the addition of a single variable of interest at a time.

The dependent variable—social network size—was log transformed. Visual inspection of residuals indicated an approximately normal distribution, and formal checks of independence indicated no violations. However, checks suggested there was an issue of heteroscedasticity; therefore, a robust regression was computed using the R package “sandwich,” which provides more accurate standard errors (Zeileis et al., 2020). In the quadratic model, there was high multicollinearity between age and age^2^; therefore, this model was run with mean-centered continuous variables.

##### Friend network size

A non-linear relationship between age and friend network size was indicated in visualizations of the raw data; therefore, the full model (Model 2.1) (consisting of control variables and variables of interest) was compared with the quadratic model (Model 2.2) consisting of the full model with the addition of a polynomial term for age. Visual inspection of residuals indicated an approximately normal distribution. Formal checks of independence and heteroscedasticity indicated no violations. In the model with the quadratic term for age, there was high multicollinearity between age and age^2^; therefore, the model was run with mean-centered continuous variables.

##### Family network size

Visual inspection of residuals indicated an approximately normal distribution. Formal checks of independence and collinearity indicated no violations. However, checks for heteroscedasticity indicated a violation; therefore, robust standard errors were computed using the “sandwich” R package. The full model (Model 3.1) comprising control variables and variables of interest is presented in Table 3.

#### Change analysis

In this analysis, six models were computed. Three binomial logistic regression models investigated whether socio-communicative traits were protective against network size (social, friend, and family) decline. The outcome variable in each model was whether the social, friend, or family network size change had decreased or not (represented by a “0” for a decreased network size, and by “1” for a network size that had remained the same or increased). Each model was compared against a null model formed of the covariates (age at baseline, gender, relationship status at final wave, and general health at final wave).

## Results

### Single-Wave Analysis

#### Social network size

Model comparison indicated that model fit was improved between the null and the full model, χ²(3) = 229.93, *p* < .001 (Model 1.1 AIC = 9309; Model 1.2 AIC = 9016). Model fit was further improved between the full model and the quadratic model (full model plus a quadratic term for age), χ²(1) = 7.75, *p* < .05 (Model 1.2 AIC = 9016; Model 1.3 AIC = 9007, *f*^2^ = 0.002). The final model for the single-wave analysis of social network size was the quadratic model. Indicating that our hypothesis that socio-communicative traits are predictive of social network size in older adults is supported.

Considering single variables at a time, the addition of each variable alone to the null model improved model fit (see supplementary material). However, the addition of variables of interest to the null model at step, in the following order—extraversion, agreeableness, and verbal communication—resulted in a significant improvement for the inclusion of extraversion, χ²(1) = 184.40, *p* < .001, then agreeableness, χ²(1) = 45.32, *p* <.001, but not for the addition of verbal communication, χ²(1) = 1.07, *p* = .30. This suggests that verbal communication is not as important to social network size as extraversion or agreeableness.

The fixed effects considered in the null model accounted for 2.4% of the variance between individuals, while the quadratic model accounted for 8%. This suggests that the socio-communicative traits were important predictors of network size in comparison to the covariates and had a significant positive effect on network size in the cross-sectional analysis.

**Figure 1.**
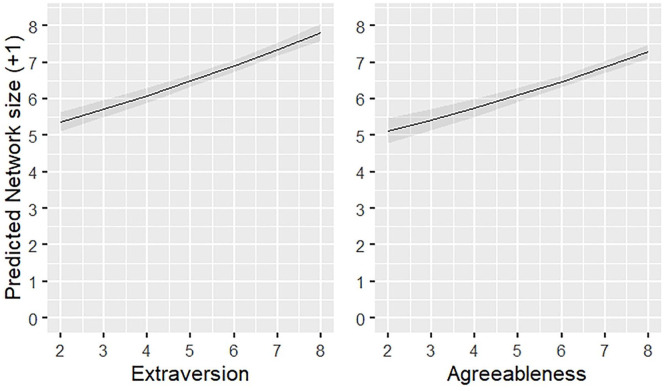
Effect plot for predicted social network size by extraversion and agreeableness based on the quadratic model (1.3) with 95% confidence intervals. *Note.* The outcome variable has been back transformed from the log-scale; however, a constant of 1 was added to the raw data as a constant to allow log-transformation of zero responses; therefore, the network size presented here is the original numerical response +1.

The standardized beta coefficients indicate that extraversion had the greatest effect on network size, followed by agreeableness and then gender (Table 2). Higher extraversion and agreeableness scores as well as being female were associated with larger social networks. In the final model, accounting for all other variables a one-unit increase in either extraversion or agreeableness equated to an increase in network size of 0.06, whereas for verbal communication a one-unit increase resulted in a network size increase of 0.01 (see supplementary material for calculation); these trends are presented in Figure 1. Verbal communication had a weaker association with network size compared with extraversion and agreeableness, but the effect was equivalent to the association between network size and relationship status. Married or cohabiting participants, and those of higher socioeconomic status, had larger network sizes. The non-linear relationship between network size and age is characterized by smaller network sizes in the youngest and oldest participants.

**Table 2.** Cross-Sectional Analyses: Linear Regression Model Estimates for Predictors of Social Network Size.

Predictors	Null model (Model 1.1)					Quadratic model (Model 1.3)				
	Social network size (log)					Social network size (log)				
	*B*	*SE B*	β	CI	*p*	*B*	*SE B*	β	CI	*p*
Age	0.002	0.001	0.03	[−0.0003, 0.004]	.095	0.003	0.001	0.05	[0.002, 0.004]	<.001
Gender	0.14	0.02	0.12	[0.11, 0.18]	<.001	0.10	0.01	0.09	[0.09, 0.11]	<.001
Relationship status	−0.04	0.02	−0.03	[−0.08, 0.003]	.033	−0.03	0.01	−0.02	[−0.04, 0.01]	<.001
General health	0.04	0.01	0.08	[0.03, 0.06]	<.001	0.02	0.004	0.03	[0.008, 0.02]	<.001
Socioeconomic status	0.01	0.01	0.04	[0.001, 0.02]	.026	0.01	0.002	0.05	[0.01, 0.02]	<.001
Age^2^						−0.0003	0.0001	−0.05	[−0.0004, 0.0002]	<.001
Agreeableness						0.06	0.004	0.12	[0.06, 0.07]	<.001
Extraversion						0.06	0.003	0.16	[0.06, 0.07]	<.001
Verbal communication						0.01	0.005	0.02	[0.002, 0.02]	.017
Model information										
*N*	5,202					5,202				
*R*^2^	.024					.080				
AIC	9309					9007				
Residual Dev	1739 (*df* = 5196)					1638 (*df* = 5193)				

*Note. B* = beta estimates; CI = confidence interval; β = standardized beta estimates; AIC = Akaike information criterion.

To attain the local effect size for each predictor, we subtracted one predictor at a time from the quadratic model (Model 1.3). The results indicated that extraversion and agreeableness had the greatest effect on network size compared with the other variables, although these effects are rather small (extraversion *f*^2^ = 0.02; agreeableness *f*^2^ = 0.01). However, the effect of extraversion on social network size in this study is similar to the association found between extraversion and support and sympathy group size (both *f*^2^ = 0.04) found in previous research (Pollet et al., 2011).

#### Friend network size and family network size

For friend network size, model comparison showed the model with a quadratic term for age (Model 2.2) had a superior fit to the model with a linear term for age (Model 2.1), χ²(1) = 9.63, *p* < .05 (AIC: 8161 and 8151, respectively). Final models for each network type are presented in Table 3.

**Table 3.** Cross-Sectional Analyses: Linear Mixed Effects Regression Model Estimates for Predictors of Friend and Family Social Network Sizes.

Predictors	Friend network model (Model 2.2)					Family network model (Model 3.1)				
	Friend network size (log)					Family network size (log)				
	*B*	*SE B*	β	CI	*p*	*B*	*SE B*	β	CI	*p*
Age	0.002	0.001	0.03	[−0.0003, 0.005]	.084	0.01	0.006	0.08	[−0.007, 0.02]	.429
Age^2^	−0.0004	0.0001	−0.06	[−0.001, −0.0001]	.002					
Gender	−0.01	0.02	−0.01	[−0.05, 0.03]	.515	0.13	0.01	0.11	[0.11, 0.14]	<.001
Relationship status	0.07	0.02	0.06	[0.03, 0.11]	<.001	−0.11	0.01	−0.09	[−0.12, −0.10]	<.001
General health	−0.001	0.01	−0.001	[−0.02, 0.02]	.933	0.003	0.004	0.006	[−0.004, 0.01]	.399
Socioeconomic status	0.02	0.005	0.08	[0.02, 0.03]	<.001	−0.01	0.002	−0.03	[−0.01, −0.004]	<.001
Agreeableness	0.05	0.01	0.08	[0.03, 0.07]	<.001	0.06	0.004	0.11	[0.05, 0.06]	<.001
Extraversion	0.08	0.01	0.19	[0.06, 0.09]	<.001	0.02	0.003	0.06	[0.02, 0.03]	<.001
Verbal communication	0.004	0.01	0.01	[−0.02, 0.03]	.770	0.007	0.01	0.01	[−0.003, 0.02]	.199
Model information										
*N*	4,587					5,050				
*R*^2^	.064					.051				
AIC	8151					8520				
Residual Dev	1499					1522				

*Note. B* = beta estimates; CI = confidence interval; β = standardized beta estimates; AIC = Akaike information criterion.

**Figure 2.**
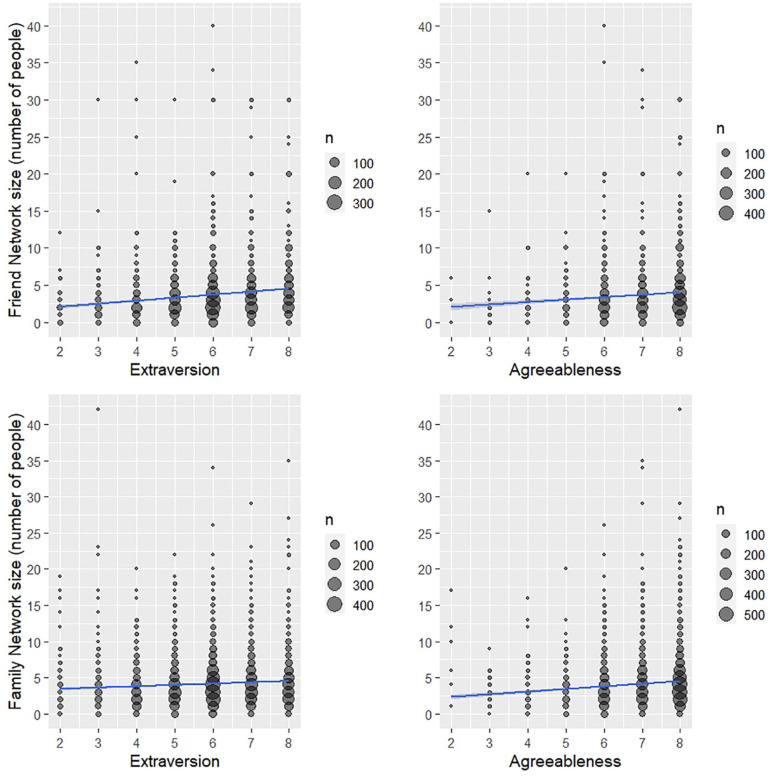
Friend and family network size by extraversion and agreeableness. *Note.* Count data are presented; the size of the datapoint indicates the number of respondents. One datapoint is not presented in Figure 2 (a participant with a reported family network size of 59) due to reducing the clarity of the figure.

Analysis of friend and family network sizes separately indicated that friend networks are more likely to be influenced by extraversion than family networks. A one-unit increase of extraversion equated to an increase in friend network size of 0.08 members and an increase in family network size of 0.02. Whereas for agreeableness and verbal communication, the relationships with friend and family network sizes were similar. In Figure 2, the stronger effect of extraversion on friend (*f*^2^ = 0.02) compared with family networks (*f*^2^ = 0.002), and the similar effect of agreeableness on friend and family network sizes (*f*^2^ = 0.01 for each) can be seen.

The predictors accounted for 6.4% of the variance between participants for friend network size and a lesser 5.1% of the variance for family network size. For friend network size, extraversion was the best predictor, followed by agreeableness and socioeconomic status. Whereas for family network size, agreeableness and gender were the best predictors, followed by relationship status and age, then extraversion.

There were some noteworthy differences in the control variables between friend and family networks. Being single, compared with married or cohabiting, had a positive relationship with friend network size but a negative relationship with family network size. The latter could be due to acquiring shared family networks from partners/spouses as well as an increased likelihood of having had children. Gender was more important to family than friend networks with female participants having significantly larger family networks than males, whereas for friend networks there was less discrepancy between genders.

As with social network size, friend network size had a non-linear relationship with age. This relationship was characterized by a greater number of friends for those in mid-later life and fewer friends for those in early and late-later life.

### Change Analysis

As can be seen in Table 4, over the 14-year period, a similar number of participants’ social network size decreased as increased, whereas a greater number of participants had their friend network increase and their family network decrease.

**Table 4.** Count for the Direction of Network Size Change for Social, Friend, and Family Network Size Change.

	Direction of network size change		
Network Type	Decreased	No change	Increased
Social	648	185	678
Friend	409	270	579
Family	575	349	509

For social network size change, model comparison indicated that model fit was not improved between the null and the full model, χ²(3) = 4.50, *p =* .21 (null AIC = 2065; full AIC = 2067). The same was true for friend and family network size change; the null models for each were not improved by the addition of the socio-communicative traits, χ²(3) = 1.57, *p=* .67 (null AIC = 1585, full AIC = 1590); χ²(3) = 7.04, *p =* .07 (null AIC = 1935, full AIC = 1934). Model results are presented in Table 5. Our hypothesis that variables found to be important predictors of social network size in the cross-sectional study would also predict social network size change is therefore not supported and the null hypothesis is accepted.

**Table 5.** Change Analyses: Binomial Regression Results for First–Last Change in Social, Friend, And Family Network Size Change.

Predictors	Social network size change			Friend network size change			Family network size change		
	Odds ratio (CI)	*SE*	*p*	Odds ratio (CI)	*SE*	*p*	Odds ratio (CI)	*SE*	*p*
Age at baseline	0.99 [0.97, 1.01]	0.01	.222	0.97 [0.95, 0.99]	0.01	.008	0.99 [0.97, 1.00]	0.01	.121
Gender	0.85 [0.68, 1.06]	0.10	.153	0.86 [0.66, 1.11]	0.11	.245	0.87 [0.69, 1.09]	0.10	.216
Relationship at close	1.07 [0.84, 1.35]	0.13	.594	1.10 [0.84, 1.45]	0.15	.506	1.10 [0.86, 1.41]	0.14	.453
General health at close	1.07 [0.97, 1.18]	0.06	.188	1.07 [0.95, 1.21]	0.07	.270	1.01 [0.91, 1.12]	0.05	.870
Agreeableness	0.92 [0.83, 1.03]	0.05	.141	0.93 [0.82, 1.05]	0.06	.263	0.94 [0.84, 1.05]	0.05	.302
Extraversion	1.09 [0.99, 1.19]	0.05	.071	1.04 [0.93, 1.15]	0.06	.521	1.06 [0.97, 1.17]	0.05	.187
Verbal communication	0.94 [0.82, 1.08]	0.07	.381	1.04 [0.88, 1.22]	0.09	.672	0.85 [0.73, 0.98]	0.06	.027
Model information									
Observations	1511			1258			1433		
*R*^2^ Tjur	.009			.010			.009		
AIC	2066.680			1589.806			1933.568		

*Note.* CI = confidence interval; AIC = Akaike information criterion.

The binomial regression models indicate that socio-communicative traits, as well as the control variables included in these analyses, were poor predictors of whether network sizes decreased or not, accounting for 1% or less of the variance between individuals.

Verbal communication had a negative relationship with the odds of having family network size decrease over time. Meaning that those who had scored higher for verbal communication had greater odds of family network size decline—A one-unit increase in verbal communication increased the odds of the loss of at least one family network member by a factor of 1.18. This could possibly be due to those with poorer communication skills requiring instrumental help from close family members, leading to more time spent with, and closer relationships with, those relatives.

## Discussion

In this research, greater extraversion and agreeableness were associated with larger social network sizes. These findings remained intact once other variables such as age, gender, and general health were controlled. Together, extraversion, agreeableness, and verbal communication accounted for more of the between-person variance in network size than all other examined variables. Extraversion and agreeableness were positively related to social, friend, and family network sizes, though extraversion appears to be more influential for friend than family network size, while agreeableness had a similar effect on both kin and friend network size. The findings of this research offer support to and extend the social brain hypothesis, as similarly to cognitive social skills, socio-communicative traits are related to social network size.

Extraversion and social network size have been linked in the literature in numerous studies. Within younger samples, the relationship between these variables appears to be reliably positive, whereas the evidence from studies with older adults has produced varied results. The results from the current research points to a similarly positive relationship between extraversion and social network size in older adults as has been found in younger samples. The conflicting findings from previous research with older adults may be due to differences between research samples. First, the sample size in this research is somewhat larger than many prior studies. For example, Iveniuk’s (2019) research found only a weak tie between extraversion and social network size in a sample of 2,261 participants, which is approximately half the sample size of the cross-sectional analysis in the present study. Second, studies with older adults suffer from a lack of classification of “later life”; there is no absolute or agreed age at which someone enters “later life” or is classed as an “older adult.” In the present study, the youngest participants were 50 years old, whereas in other studies different age limits are set (e.g., youngest age was 62 in Iveniuk, 2019).

The finding of this research that more extraverted or agreeable individuals have larger social networks is somewhat intuitive. Both extraversion and agreeableness are described by traits linked to social interaction or behaviors. Extraverts are often characterized as being outgoing, thriving in groups and enjoying the attention of others among other more proximal characteristics such as being enthusiastic or lively (McCrae & John, 1992). Extraverts are also more likely to engage in networking behaviors such as socializing or maintaining contacts (Forret & Dougherty, 2001) and may be more likely to develop new friendships or seek new connections (Asendorpf & Wilpers, 1998; Selden & Goodie, 2018). This is supported by the findings in this study that extraversion had a greater impact on friendship compared with family network size. Agreeable individuals are often described as being warm, friendly, and kind. Some researchers propose that agreeableness can be understood in terms of motivation to maintain smooth social relationships and is strongly related to social behaviors such as helping behavior, conflict resolution, and cooperation (Tobin & Gadke, 2015). Research indicates that while extraversion appears to be a driver of relationship formation, agreeableness may be the trait that is pivotal for the maintenance (rather than the establishment) of relationships (Harris & Vazire, 2016).

Age was a significant predictor of social network size and for friend network size. Age had a non-linear relationship with both social network size and friend network size, suggesting that for friendship ties, at least, there could be a period of both growth and reduction of ties in this age group. In this study, individuals with the greatest social network size were those of approximately 65–75 years of age. The average age of retirement in the United Kingdom is 63.9 for women and 65.1 for men (Department for Work & Pensions [DWP], 2018); therefore, the findings of this research could be indicative of a network size growth spurt in the years following retirement from additional friendship ties. This is contrary to previous studies which have found retirement does not relate to changes in overall social network size (Fletcher, 2014; Van Tilburg, 1998). However, the current study investigates the size of one’s close social network rather than a global network size (which is more likely to include workplace acquaintances). Therefore, it is possible that the larger network sizes found in this sample around retirement age is related to having more time to invest in close relationships.

Lifespan studies report vastly different accounts of the direction and size of the network size change over time. The findings of some studies suggest network size gradually decreases in later life, while others report that overall network size does not change or may actually increase in later life (Cornwell et al., 2014; Van Tilburg, 1998; Wrzus et al., 2013). Our findings suggest that network sizes remain largely stable over time: The mean social, friend, and family network size change was close to zero. There was not strong support for a relationship between socio-communicative traits and network size change. The addition of socio-communicative traits to the models did not improve the models of network size change. However, it should be noted that the covariates were also not strong predictors of network size change. This is somewhat surprising as previous longitudinal research has found demographic variables such as gender and health to be significant predictors of network size change (English & Carstensen, 2014). Of the covariates, age at baseline had a significant relationship with friend network size change; those who were older at baseline were more likely to have had their friend network size decline by the final wave. However, this was not the case for family network size change or social network size change.

There could be a few reasons why we do not see any influence of socio-communicative traits on social network size change. First, in the ELSA, self-reported traits that formed our measures of personality and verbal communication were only collected at Wave 5. Consequently, this study cannot look at the time-varying effects of these traits, which could be particularly important as extraversion and verbal communication decrease with age. For example, poor health in old age is related to changes in personality traits, including decreases in agreeableness and extraversion (Kornadt et al., 2018). It is also the case that the relationship between maintenance behaviors, such as time spent with friends, and extraversion becomes less significant in later life (Wrzus et al., 2016). Therefore, the positive impact of extraversion on social network size at a fixed time point may not translate into an association with network size change. Second, it is possible that the positive influence of extraversion and agreeableness on network size are counteracted by other individual differences or contextual events. For example, the Differential Investment in Resources Model proposes that individual changes in capacity and motivation may alter the amount of time and energy one may invest into social ties (Fiori et al., 2020). Furthermore, extrinsic reasons for network size change cannot be identified in this research, events such as the death of close friend or family member will result in a change in network size, but that variation has not been captured.

Communication, in its many forms, is vital for social interaction. Evolutionary theory proposes that communicative ability may be strongly connected to the number and quality of potential relationships, in that species with superior socio-cognitive skills are capable of maintaining more social connections (Dunbar, 1998). Within humans, research on the relationship between communication and social network size has been limited predominantly to research in clinical populations—with diverse communicative deficiencies. Quite consistently this research has highlighted that those individuals with communicative disorders are at risk of having smaller social networks (Palmer et al., 2016). This research attempted to study communicative ability in a typical (non-clinical) population of older adults. In this research, this variable had a weak association with social, friend, and family network size. While this does suggest that talkativeness is not as important to social network size as extraversion and agreeableness, there are some measurement issues that could influence this result. In this study, verbal communication was derived from a single item in the ELSA data set, self-reported “talkativeness,” which implies both ability and preference to communicate. In an online concurrent validity study, we found that this measure had a moderate–strong correlation with communication competence, but only a small association with communicative preference, which suggests that verbal communication may be more analogous to self-assessed communicative ability and does not capture the individual differences in trait communicative preference. Perhaps preference to communicate with others is more important than communicative ability to the formation and maintenance of social relationships, which could explain why we have found only a weak relationship between verbal communication and social network size here. Future work could address the question of whether communicative preference relates to social network size or the outcomes of social interactions.

A limitation of this study is that we consider only the inner layers of an individual’s social network—the support clique and the sympathy group. The size of one’s outer network, formed of weaker ties, can also impact well-being, and reported happiness. A greater number of interactions with weak ties can positively impact happiness and sense of belonging (Sandstrom & Dunn, 2014). As can having a larger number of weak ties; researchers have found that having more weak ties present in a social network can positively influence emotional well-being over time in older adults (Huxhold et al., 2020). Extraversion has been shown to relate to having a larger number of weak ties in the outer layer of the network in adult samples (Pollet et al., 2011). Therefore, socio-communicative traits may well be consequential to size of the outer layers of older adults’ social network, but this could not be addressed in this study.

This study, like many household panel surveys, is limited by the reliance on self-reported data. Most ELSA data are collected via Computer-Assisted Personal Interviewing (CAPI), where an interviewer verbally asks the survey questions and records the responses, allowing for the inclusion of participants who may be unable or uncomfortable using a computer. While the interviewer ensures ease for the participants and inclusivity, the presence of an interviewer may reduce the sense of anonymity, which may affect some responses. Furthermore, the ELSA has not used standardized measures to assess personality traits and verbal communication; these are derived from traits responses taken at Wave 5. Consequently, it is difficult to generalize to studies that use other methods of quantifying these traits. There are also limitations of using single-item and two-item measures. The internal consistency of the Extraversion and Agreeableness measures in this study was acceptable, but the Verbal Communication measure consisted of a single item. The value of the research would be improved by using previously validated measures with a wealth of reliability information. A future body of research by this research team will utilize varied measures and techniques to investigate the link between socio-communicative traits and social network size.

### Conclusion

Network size has been found to be a protective factor in health, well-being, and cognition. It is vital, therefore, to understand the factors that predict network size. In this study, we found that extraversion and agreeableness predict older adults’ social network sizes, overall, and at the family and friend network level. Supporting the evolutionary theory that socio-communicative abilities may be associated with larger social networks. However, socio-communicative traits were not protective against network size change. This study illustrates the importance of considering individual differences in social network research.

## Supplemental Material

sj-docx-1-psp-10.1177_01461672221078664 – Supplemental material for Personality Traits Predict Social Network Size in Older Adults Click here for additional data file. Supplemental material, sj-docx-1-psp-10.1177_01461672221078664 for Personality Traits Predict Social Network Size in Older Adults by Jasmine Rollings, Jérôme Micheletta, Darren Van Laar and Bridget M. Waller in Personality and Social Psychology Bulletin

## References

[bibr1-01461672221078664] Adams R. G. Blieszner R. (1994). An integrative conceptual framework for friendship research. Journal of Social and Personal Relationships, 11(2), 163–184. 10.1177/0265407594112001

[bibr2-01461672221078664] Asendorpf J. B. Wilpers S. (1998). Personality effects on social relationships. Journal of Personality and Social Psychology, 74(6), 1531–1544. 10.1037/0022-3514.74.6.1531

[bibr3-01461672221078664] Bates D. Maechler M. Bolker B. Walker S. (2015). Fitting linear mixed-effects models using lme4. Journal of Statistical Software, 67, 1–48.

[bibr4-01461672221078664] Bickart K. C. Wright C. I. Dautoff R. J. Dickerson B. C. Barrett L. F. (2011). Amygdala volume and social network size in humans. Nature Neuroscience, 14(2), 163–164. 10.1038/nn.2724 21186358 PMC3079404

[bibr5-01461672221078664] Blood G. W. Simpson K. C. Raimondi S. C. Dineen M. Kauffman S. M. Stagaard K. A. (1994). Social support in laryngeal cancer survivors. American Journal of Speech-Language Pathology, 3(1), 37–44. 10.1044/1058-0360.0301.37

[bibr6-01461672221078664] Buys C. J. Larson K. L. (1979). Human sympathy groups. Psychological Reports, 45(2), 547–553. 10.2466/pr0.1979.45.2.547 1454924

[bibr7-01461672221078664] Carstensen L. L. (2021). Socioemotional selectivity theory: The role of perceived endings in human motivation. The Gerontologist, 61(8), 1188–1196. 10.1093/GERONT/GNAB116 34718558 PMC8599276

[bibr8-01461672221078664] Chan Y. K. Lee R. P. L. (2006). Network size, social support and happiness in later life: A comparative study of Beijing and Hong Kong. Journal of Happiness Studies, 7(1), 87–112. 10.1007/s10902-005-1915-1

[bibr9-01461672221078664] Cho J. H.-J. Olmstead R. Choi H. Carrillo C. Seeman T. E. Irwin M. R. (2019). Associations of objective versus subjective social isolation with sleep disturbance, depression, and fatigue in community-dwelling older adults. Aging & Mental Health, 23(9), 1130–1138. 10.1080/13607863.2018.1481928 30284454 PMC6447478

[bibr10-01461672221078664] Cornwell B. Schumm L. P. Laumann E. O. Kim J. Kim Y.-J. (2014). Assessment of social network change in a National Longitudinal Survey. The Journals of Gerontology, Series B: Psychological Sciences and Social Sciences, 69(Suppl. 2), S75–S82. 10.1093/geronb/gbu037 PMC4303098 25360026

[bibr11-01461672221078664] Davidson B. Howe T. Worrall L. Hickson L. Togher L. (2008). Social participation for older people with Aphasia: The impact of communication disability on friendships. Topics in Stroke Rehabilitation, 15(4), 325–340. 10.1310/tsr1504-325 18782736

[bibr12-01461672221078664] Department for Work & Pensions. (2018). Economic labour market status of individuals aged 50 and over, trends over time: October 2018 (experimental). https://assets.publishing.service.gov.uk/government/uploads/system/uploads/attachment_data/file/747715/economic-labour-market-status-of-individuals-aged-50-and-over-oct-2018.pdf

[bibr13-01461672221078664] Dunbar R. I. M. (1993). Coevolution of neocortical size, group size and language in humans. Behavioral and Brain Sciences, 16(4), 681–694. 10.1017/S0140525X00032325

[bibr14-01461672221078664] Dunbar R. I. M. (1998). The social brain hypothesis. Evolutionary Anthropology: Issues, News, and Reviews, 6(5), 178–190. 10.1002/(SICI)1520-6505(1998)6:5<178::AID-EVAN5>3.0.CO;2-8

[bibr15-01461672221078664] Dunbar R. I. M. Shultz S. (2007). Evolution in the social brain. Science, 317(5843), 1344–1347. 10.1126/science.1145463 17823343

[bibr16-01461672221078664] Dunbar R. I. M. Spoors M. (1995). Social networks, support cliques, and kinship. Human Nature, 6(3), 273–290. 10.1007/BF02734142 24203093

[bibr17-01461672221078664] English T. Carstensen L. L. (2014). Selective narrowing of social networks across adulthood is associated with improved emotional experience in daily life. International Journal of Behavioral Development, 38(2), 195–202. 10.1177/0165025413515404 24910483 PMC4045107

[bibr18-01461672221078664] Fletcher J. M. (2014). Late life transitions and social networks: The case of retirement. Economics Letters, 125(3), 459–462. 10.1016/J.ECONLET.2014.10.004

[bibr19-01461672221078664] Fiori K. Windsor T. Huxhold O . (2020). Rethinking social relationships: The differential investment of resources model of social development. Innovation in Aging, 4, 406–407. 10.1093/GERONI/IGAA057.1309 PMC8978474 35001730

[bibr20-01461672221078664] Forret M. L. Dougherty T. W. (2001). Correlates of networking behavior for managerial and professional employees. Group & Organization Management, 26, 283–311. https://journals.sagepub.com/doi/pdf/10.1177/1059601101263004

[bibr21-01461672221078664] Fox J. Weisberg S. (2011). An {R} companied to applied regression (2nd ed.). SAGE.

[bibr22-01461672221078664] Freeberg T. M. Gentry K. E. Sieving K. E. Lucas J. R. (2019). On understanding the nature and evolution of social cognition: A need for the study of communication. Animal Behaviour, 155, 279–286. 10.1016/j.anbehav.2019.04.014

[bibr23-01461672221078664] Gosling S. D. Rentfrow P. J. Swann W. B. (2003). A very brief measure of the Big-Five personality domains. Journal of Research in Personality, 37(6), 504–528. 10.1016/S0092-6566(03)00046-1

[bibr24-01461672221078664] Harris K. Vazire S. (2016). On friendship development and the Big Five personality traits. Social and Personality Psychology Compass, 10(11), 647–667. 10.1111/spc3.12287

[bibr25-01461672221078664] Hill R. A. Dunbar R. I. M. (2003). Social network size in humans. Human Nature, 14(1), 53–72. 10.1007/s12110-003-1016-y 26189988

[bibr26-01461672221078664] Holt-Lunstad J. Smith T. B. Baker M. Harris T. Stephenson D. (2015). Loneliness and social isolation as risk factors for mortality. Perspectives on Psychological Science, 10(2), 227–237. 10.1177/1745691614568352 25910392

[bibr27-01461672221078664] Holt-Lunstad J. Smith T. B. Layton J. B. (2010). Social relationships and mortality risk: A meta-analytic review. PLOS Medicine, 7(7), Article e1000316. 10.1371/journal.pmed.1000316 PMC2910600 20668659

[bibr28-01461672221078664] Huxhold O. Fiori K. L. Webster N. J. Antonucci T. C . (2020). The strength of weaker ties: An underexplored resource for maintaining emotional well-being in later life. The Journals of Gerontology: Series B, 75(7), 1433–1442. 10.1093/GERONB/GBAA019 PMC7424273 32055856

[bibr29-01461672221078664] Huxhold O. Fiori K. L. Windsor T. D. (2013). The dynamic interplay of social network characteristics, subjective well-being, and health: The costs and benefits of socio-emotional selectivity. Psychology and Aging, 28(1), 3–16. 10.1037/a0030170 23066804

[bibr30-01461672221078664] Iveniuk J. (2019). Social networks, role-relationships, and personality in older adulthood. The Journals of Gerontology, Series B: Psychological Sciences and Social Sciences, 74(5), 815–826. 10.1093/geronb/gbx120 29529263 PMC6566325

[bibr31-01461672221078664] Jensen-Campbell L. A. Adams R. Perry D. G. Workman K. A. Furdella J. Q. Egan S. K. (2002). Agreeableness, extraversion, and peer relations in early adolescence: Winning friends and deflecting aggression. Journal of Research in Personality, 36(3), 224–251. 10.1006/JRPE.2002.2348

[bibr32-01461672221078664] Johnston R. Jones K. Manley D. (2018). Confounding and collinearity in regression analysis: A cautionary tale and an alternative procedure, illustrated by studies of British voting behaviour. Quality and Quantity, 52(4), 1957–1976. 10.1007/s11135-017-0584-6 29937587 PMC5993839

[bibr33-01461672221078664] Kanai R. Bahrami B. Roylance R. Rees G. (2012). Online social network size is reflected in human brain structure. Proceedings of the Royal Society B: Biological Sciences, 279(1732), 1327–1334. 10.1098/rspb.2011.1959 PMC3282379 22012980

[bibr34-01461672221078664] Kornadt A. E. Hagemeyer B. Neyer F. J. Kandler C. (2018). Sound body, sound mind? The interrelation between health change and personality change in old age. European Journal of Personality, 32(1), 30–45. 10.1002/per.2135

[bibr35-01461672221078664] Lumlet T. (2004). Analysis of complex survey samples. Journal of Statistical Software, 9(1), 1–19.

[bibr36-01461672221078664] McCrae R. R. John O. P. (1992). An introduction to the Five-Factor model and its applications. Journal of Personality, 60(2), 175–215. 10.1111/j.1467-6494.1992.tb00970.x 1635039

[bibr37-01461672221078664] McCrae R. R. Terracciano A. (2005). Universal features of personality traits from the observer’s perspective: Data from 50 cultures. Journal of Personality and Social Psychology, 88(3), 547–561. 10.1037/0022-3514.88.3.547 15740445

[bibr38-01461672221078664] McCroskey J. C. McCroskey L. L. (1988). Self-report as an approach to measuring communication competence. Communication Research Reports, 5(2), 108–113. 10.1080/08824098809359810

[bibr39-01461672221078664] McCroskey J. C. Richmond V. P. (1987). Willingness to communicate. In McCroskey J. C. Daly J. A. (Eds.), Personality and interpersonal communication (pp. 119–131). SAGE.

[bibr40-01461672221078664] Molho C. Roberts S. G. B. de Vries R. E. Pollet T. V. (2016). The six dimensions of personality (HEXACO) and their associations with network layer size and emotional closeness to network members. Personality and Individual Differences, 99, 144–148. 10.1016/j.paid.2016.04.096

[bibr41-01461672221078664] Oldfield Z. Rogers N. Phelps A. Blake M. Steptoe A. Oskala A. Marmot M. Clemens S. Nazroo J. Banks J. (2020). English longitudinal study of ageing: Waves 0-9, 1998-2019 [Data collection] (SN:5050, 33rd ed.). UK Data Service. https://doi.org/doi.org/10.5255/UKDA-SN-5050-24

[bibr42-01461672221078664] Palmer A. D. Newsom J. T. Rook K. S. (2016). How does difficulty communicating affect the social relationships of older adults? An exploration using data from a national survey. Journal of Communication Disorders, 62, 131–146. 10.1016/j.jcomdis.2016.06.002 27420152 PMC4968942

[bibr43-01461672221078664] Pollet T. V. Roberts S. G. B. Dunbar R. I. M. (2011). Extraverts have larger social network layers: But do not feel emotionally closer to individuals at any layer. Journal of Individual Differences, 32(3), 161–169. 10.1027/1614-0001/a000048

[bibr44-01461672221078664] Rafnsson S. B. Shankar A. Steptoe A. (2015). Longitudinal influences of social network characteristics on subjective well-being of older adults: Findings from the ELSA study. Journal of Aging and Health, 27(5), 919–934. 10.1177/0898264315572111 25804898

[bibr45-01461672221078664] R Core Team. (2018). R: A language and environment for statistical. https://www.r-project.org/

[bibr46-01461672221078664] Roberts S. G. B. Dunbar R. I. M. (2011). The costs of family and friends: An 18-month longitudinal study of relationship maintenance and decay. Evolution and Human Behavior, 32(3), 186–197. 10.1016/J.EVOLHUMBEHAV.2010.08.005

[bibr47-01461672221078664] Roberts S. G. B. Dunbar R. I. M. Pollet T. V. Kuppens T . (2009). Exploring variation in active network size: Constraints and ego characteristics. Social Networks, 31(2), 138–146. 10.1016/j.socnet.2008.12.002

[bibr48-01461672221078664] Roberts S. G. B. Wilson R. Fedurek P. Dunbar R. I. M. (2008). Individual differences and personal social network size and structure. Personality and Individual Differences, 44(4), 954–964. 10.1016/j.paid.2007.10.033

[bibr49-01461672221078664] Rubin R. B. Martin M. M. (1994). Development of a measure of interpersonal communication competence. Communication Research Reports, 11(1), 33–44. 10.1080/08824099409359938

[bibr50-01461672221078664] Sandstrom G. M. Dunn E. W . (2014). Social interactions and well-being: The surprising power of weak ties. Personality and Social Psychology Bulletin, 40(7), 910–922. 10.1177/0146167214529799 24769739

[bibr51-01461672221078664] Santini Z. I. Koyanagi A. Tyrovolas S. Mason C. Haro J. M. (2015). The association between social relationships and depression: A systematic review. Journal of Affective Disorders, 175, 53–65. 10.1016/J.JAD.2014.12.049 25594512

[bibr52-01461672221078664] Schutter N. Koorevaar L. Holwerda T. J. Stek M. L. Dekker J. Comijs H. C. (2019). “Big Five” personality characteristics are associated with loneliness but not with social network size in older adults, irrespective of depression. International Psychogeriatrics, 32(1), 53–63. 10.1017/S1041610219000231 30968789

[bibr53-01461672221078664] Selden M. Goodie A. S . (2018). Review of the effects of five factor model personality traits on network structures and perceptions of structure. Social Networks, 52, 81–99. 10.1016/J.SOCNET.2017.05.00

[bibr54-01461672221078664] Selya A. S. Rose J. S. Dierker L. C. Hedeker D. Mermelstein R. J. (2012). A practical guide to calculating Cohen’s f 2, a measure of local effect size, from PROC MIXED. Frontiers in Psychology, 3, Article 111. 10.3389/FPSYG.2012.00111/BIBTEX PMC3328081 22529829

[bibr55-01461672221078664] Steptoe A. Breeze E. Banks J. Nazroo J. (2013). Cohort profile: The English Longitudinal Study of Ageing. International Journal of Epidemiology, 42(6), 1640–1648. 10.1093/ije/dys168 23143611 PMC3900867

[bibr56-01461672221078664] Sutcliffe A. Dunbar R. I. M. Binder J. Arrow H . (2012). Relationships and the social brain: Integrating psychological and evolutionary perspectives. British Journal of Psychology, 103(2), 149–168. 10.1111/J.2044-8295.2011.02061.X 22506741

[bibr57-01461672221078664] Tobin R. M. Gadke D. L. (2015). Agreeableness. In Wright J. D. (Ed.), International Encyclopedia of the social & behavioral sciences (2nd ed., pp. 463–470). Elsevier. 10.1016/B978-0-08-097086-8.25044-7

[bibr58-01461672221078664] Tov W. Nai Z. L. Lee H. W. (2016). Extraversion and agreeableness: Divergent routes to daily satisfaction with social relationships. Journal of Personality, 84(1), 121–134. 10.1111/jopy.12146 25345667

[bibr59-01461672221078664] Van Tilburg T . (1995). Delineation of the social network and differences in network size. In Knipscheer C. P. M. de Jong Gierveld J. Van Tilburg T. Dykstra P. A. (Eds.), Living arrangements and social networks of older adults (pp. 83–96). VU University Press. https://www.researchgate.net/publication/227944518.

[bibr60-01461672221078664] Van Tilburg T . (1998). Losing and gaining in old age: Changes in personal network size and social support in a four-year longitudinal study. The Journals of Gerontology, Series B: Psychological Sciences and Social Sciences, 53(6), S313–S323. 10.1093/geronb/53B.6.S313 9826973

[bibr61-01461672221078664] Wrzus C. Hänel M. Wagner J. Neyer F. J. (2013). Social network changes and life events across the life span: A meta-analysis. Psychological Bulletin, 139(1), 53–80. 10.1037/a0028601 22642230

[bibr62-01461672221078664] Wrzus C. Wagner G. G. Riediger M. (2016). Personality-situation transactions from adolescence to old age. Journal of Personality and Social Psychology, 110(5), 782–799. 10.1037/PSPP0000054 26167797

[bibr63-01461672221078664] Yorkston K. M. Bourgeois M. S. Baylor C. R. (2010). Communication and aging. Physical Medicine and Rehabilitation Clinics of North America, 21(2), 309–319. 10.1016/J.PMR.2009.12.011 20494279 PMC3074568

[bibr64-01461672221078664] Zeileis A. Köll S. Graham N. (2020). Various versatile variances: An object-oriented implementation of clustered covariances in R. Journal of Statistical Software, 95(1), 1–36. 10.18637/jss.v095.i01

[bibr65-01461672221078664] Zhou W. X. Sornette D. Hill R. A. Dunbar R. I. M. (2005). Discrete hierarchical organization of social group sizes. Proceedings of the Royal Society B: Biological Sciences, 272(1561), 439–444. 10.1098/rspb.2004.2970 PMC1634986 15734699

