# Supplementary material for: Personality Traits Predict Social Network Size in Older Adults
Source: Pers Soc Psychol Bull. 2022 Apr 8;49(6):925–38. doi: 10.1177/01461672221078664 (PMC10226003; doi:10.1177/01461672221078664)
Supplement: sj-docx-1-psp-10.1177_01461672221078664 – Supplemental material for Personality Traits Predict Social Network Size in Older Adults [file sj-docx-1-psp-10.1177_01461672221078664.docx]

**Supplementary material: Personality traits predict social network size in older adults**

Contents

1. Additional information on ELSA data
2. Additional information on sample characteristics and correlation matrix
3. Measure Validation Study (trait verbal communication)
4. Single-Wave Analysis Supplementary
   1. Assumption testing
      1. Total Network Size
      2. Friend Network Size
      3. Family Network Size
   2. Additional model comparisons and extra information
      1. Total Network Size
   3. Supplementary plots
   4. Interpreting Regression Coefficients
5. Treating verbal communication as ordinal
6. Bootstrapping
7. References
8. R Packages
9. **Additional information on the ELSA data**

The ELSA sample was originally drawn from households that had participated in the Health Survey for England (HSE) from 1998-2001. This original sample, plus younger cohorts added intermittently to retain the representative sample, were interviewed every 2 years; these bi-annual interviews are known as waves. The ELSA provides data on mental and physical health, social engagement, individual traits, attitudes, finances, housing, and social care, although the exact content is subject to change in different waves (English Longitudinal Study of Ageing, 2019). A detailed description of sampling and data collection procedures has been outlined in previous research (Steptoe et al., 2013). For more information on the variables in this study or any other variable see ELSA’s data dictionaries (English Longitudinal Study of Ageing, 2016). All data is freely available on the UK data service (Oldfield et al., 2020).

1. **Additional information on sample characteristics and correlation matrix**

*Table. 1. Sample Characteristics for Wave 5 ELSA data (n=7505)*

|  | **Overall (N=7505)** |
| --- | --- |
| **Network size** |  |
| Mean (SD) | 7.30 (5.01) |
| Median [Min, Max] | 6.00 [0, 107] |
| **Friend Network (missing 886 – 11.8%)** |  |
| Mean (SD) | 3.69 (3.40) |
| Median [Min, Max] | 3.00 [0, 97.0] |
| **Family Network (missing 208 – 2.8%)** |  |
| Mean (SD) | 4.16 (3.11) |
| Median [Min, Max] | 4.00 [0, 59.0] |
| **Age** |  |
| Mean (SD) | 66.1 (8.59) |
| Median [Min, Max] | 65.0 [50.0, 89.0] |
| **Gender** |  |
| Male | 3324 (44.3%) |
| Female | 4181 (55.7%) |
| **Relationship** |  |
| Married/cohabiting | 5503 (73.3%) |
| Single | 2002 (26.7%) |
| **General Health** |  |
| Mean (SD) | 3.28 (1.08) |
| Median [Min, Max] | 3.00 [1.00, 5.00] |
| **Socioeconomic Status** |  |
| Mean (SD) | 5.16 (2.01) |
| Median [Min, Max] | 5.00 [1.00, 8.00] |
| **Agreeableness** |  |
| Mean (SD) | 7.04 (1.07) |
| Median [Min, Max] | 7.00 [2.00, 8.00] |
| **Extraversion** |  |
| Mean (SD) | 5.97 (1.45) |
| Median [Min, Max] | 6.00 [2.00, 8.00] |
| **Verbal Communication** |  |
| Mean (SD) | 3.06 (0.860) |
| Median [Min, Max] | 3.00 [1.00, 4.00] |

*Table. 2. Sample Characteristics for Core Members Analysis Wave 5 ELSA data (n=5202)*

|  | **Overall (N=5202)** |
| --- | --- |
| **Network size** |  |
| Mean (SD) | 7.31 (4.98) |
| Median [Min, Max] | 6 [0, 79] |
| **Friend Network (missing 615 – 11.8%)** |  |
| Mean (SD) | 3.72 (3.28) |
| Median [Min, Max] | 3 [0, 40] |
| **Family Network (missing 152 – 2.9%)** |  |
| Mean (SD) | 4.15 (3.12) |
| Median [Min, Max] | 4 [0, 59] |
| **Age** |  |
| Mean (SD) | 66.8 (8.62) |
| Median [Min, Max] | 65 [52, 89] |
| **Gender** |  |
| Male | 2194 (42.2%) |
| Female | 3008 (57.8%) |
| **Relationship** |  |
| Married/cohabiting | 3218 (61.9%) |
| Single | 1984 (38.1%) |
| **General Health** |  |
| Mean (SD) | 3.24 (1.09) |
| Median [Min, Max] | 3 [1, 5] |
| **Socioeconomic Status** |  |
| Mean (SD) | 5.11 (2.01) |
| Median [Min, Max] | 5 [1 , 8] |
| **Agreeableness** |  |
| Mean (SD) | 7.05 (1.08) |
| Median [Min, Max] | 7 [2, 8] |
| **Extraversion** |  |
| Mean (SD) | 5.95 (1.47) |
| Median [Min, Max] | 6 [2, 8] |
| **Verbal Communication** |  |
| Mean (SD) | 3.07 (0.87) |
| Median [Min, Max] | 3 [1, 4] |

*Figure. 1. Correlation Matrix for Wave 5 ELSA data (n=5202)*


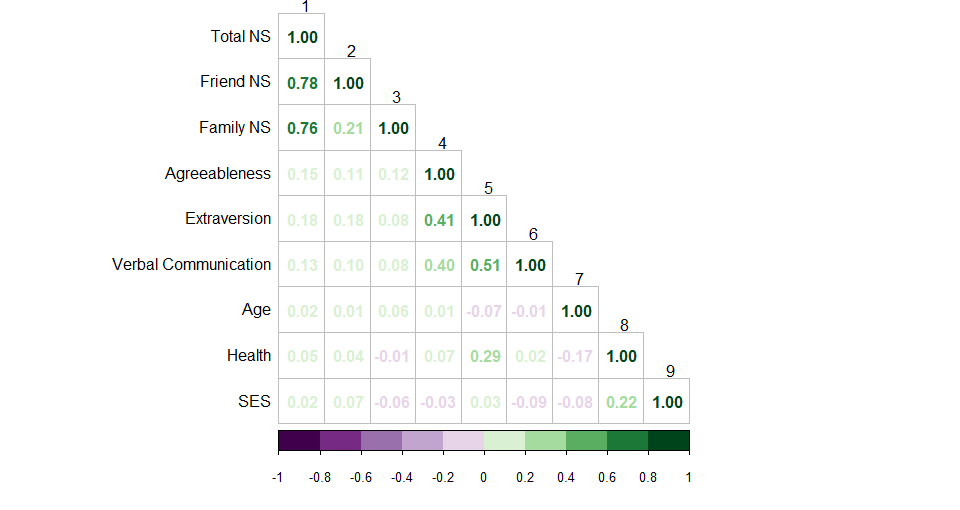


*Note.* Correlation Matrix of continuous variables using in regression models. NS= Network Size. SES = socioeconomic status

*
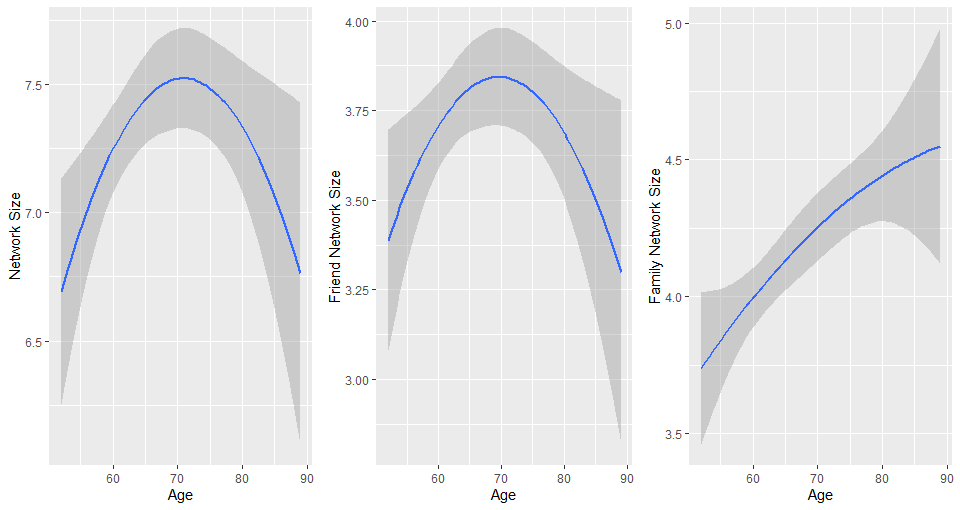
Figure. 2. Age by Total, Friend and Family Network Size with polynomial fit for age*

*Note.* Figures are on different y-axis and do not account for full range of x responses. These figures were created to aid inspection of variable relationships only.

Based on comparison of the linear and polynomial fit for age, there appears to be a linear relationship between age and family network size but for total and friend network size the relationship appears to be quadratic, see Figure 2. Model comparisons will be carried out to compare age and age^2^ in predicting total and friendship network size.

1. **Measure Validation Study**

Aim

To investigate concurrent validity between verbal communication as determined by trait talkativeness from the English Longitudinal Study of Ageing (ELSA) and communicative competence and preference.

Method

*Participants*

101 participants were recruited via *Prolific.ac*, a platform for hosting online surveys. Participants were prescreened so that the sample included participants aged 50+, fluent in English and currently residing in the UK. Participants were aged 50-76, the average age was 57.4 (*SD* = 6.23), 55% were female.

*Measures*

Verbal Communication

Participants were asked to respond on a scale from ‘Not at all’ to ‘A lot’ how ‘talkative’ they believe they are, which resulted in a score of 1 – 4. This question is identical to the one asked in the English Longitudinal Study of Ageing (ELSA). Talkativeness is used in this study as a proxy for verbal communication.

Communicative Competence

To assess communicative competence, we utlised the Self-Perceived Communication Competence Scale (SPCC) (McCroskey & McCroskey, 1988) and the Interpersonal Communication Competence Scale (short version) (ICCS-SF) (Rubin & Martin, 1994).

The SPCC consists of 12 items designed to measure one’s perceived competence in a variety of communication contexts, this is different from measuring actual communicative competence. In this study the SPCC had excellent internal reliability (Cronbach’s α = 0.94).

The ICCS-SF consists of 10 items designed to self-assess an individual’s ability to communicate in interpersonal communication settings. The ICCS-SF has shown concurrent validity with both cognitive and communication flexibility (Rubin & Martin, 1994). In this study the ICCS-SF had good internal reliability (Cronbach’s α = 0.81).

Communicative Preference

To assess preference to communicate we used to Willingness to Communicate scale (WTC; McCroskey & Richmond, 1987). The WTC measures a person's willingness to initiate interpersonal communication. The measure consists of 20 items, though eight are used to distract from the scored items. The WTC had excellent internal reliability in this study (Cronbach’s α = 0.93).

Social Network Size

Social network questions were identical to those asked in the ELSA, which were the number of children, family members (other than spouse or children) and friend’s participants ‘felt close to’, which required a numerical response. This is then summed to provide an overall social network size. Friend and family network size is established based on the single item for each social group.

Covariates

Participants were asked to report on their gender, age, and current place of residence with the UK (England, Scotland, Wales, Northern Ireland).

*Procedure*

After signing up to participate on *Prolific.ac*, participants were routed to the online survey hosted on Qualtrics. Participants provided informed consent, followed by basic demographic questions, then the WTC scale, questions regarding their social network, the ICCS-SF and finally the SPCC. Participants then reached a debrief page with details on how to contact the experimenter and the relevant complaints officer. Participants were reimbursed after successful completion of the survey (based on complete responses and correct responses to attention checks).

Results

The average score for talkativeness was 2.52 (*SD* = 1.11) out of possible high score of 4. Social network sizes ranged from 0 – 33 with a mean of 8.51 (*SD* = 5.60).

Trait verbal communication, based on self-perceived talkativeness, was associated with communication competence. Both the SPCC and the ICCS-SF had moderate-strong correlations with verbal communication (*r* = . 47, *p* < .001; *r* = . 60, *p* < .001) as well as with one another (*r* = . 62, *p* < .001). However, verbal communication had only a small correlation with communicative preference (*r* = . 26, *p* < .01).

*
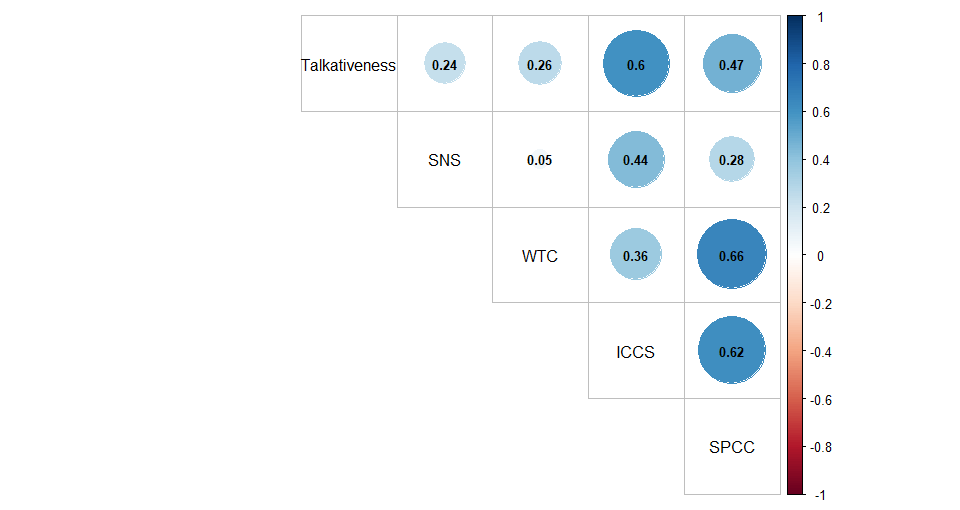
Figure. 1. Correlations between communication measures and social network size*

*Note.* SNS = Social Network Size; WTC = Willingness to Communicate; ICCS = Interpersonal Communication Competence; SPCC = Self-perceived Communication Competence.

As can be seen in Figure 1, self-perceived communicative competence (as assessed by the SPCC) and willingness to communicate (as assessed by the WTC) had small correlations with overall social network size, whilst communicative competence (as assessed by the ICCS-SF) had a moderate correlation with overall social network size.

In a multiple linear regression, the combination of SPCC (β = −.01), *t*(94) = 1.86, *p* = .07; ICCS (β = .08), *t*(94) = 4.40, *p* < .001; and WTC (β = −.003), *t*(94) = −0.56, *p* = .57, predicted 37% of the variance between individuals. This is controlling for age, gender, and whether the individual has or previously had a physical impairment that affected their communication.

Conclusion

The results indicate that the verbal communication, assessed by trait talkativeness in the ELSA, is related to interpersonal communicative ability in older adults. Trait verbal communication had a greater association with measures of communicative competence rather than communicative preference.

*References*

McCroskey, J. C., & McCroskey, L. L. (1988). Self‐report as an approach to measuring communication competence. *Communication Research Reports*, *5*(2), 108–113. https://doi.org/10.1080/08824098809359810

McCroskey, J. C., & Richmond, V. P. (1987). Willingness to communicate. In J. C. McCroskey & J. A. Daly (Eds.), *Personality and interpersonal communication* (pp. 119–131). Newbury Park, CA: Sage.

Rubin, R. B., & Martin, M. M. (1994). Development of a measure of interpersonal communication competence. *Communication Research Reports*, *11*(1), 33–44. https://doi.org/10.1080/08824099409359938

1. **Single-Wave Analysis Supplementary**
2. Assumption Testing

Using the R package, “stats” (version 4.1.0) visual inspection of model residuals was performed. The R package “performance” (version 0.7.2) was used to carry out checks to assess multicollinearity, independence, and heteroscedasticity.

- - 1. **Total Network Size**

Based on the core members dataset (*n* = 5202) and the following model:

Network size ~ age + sex + relationship + health + SES +Agreeableness + Extraversion + Verbal Communication, (weighting = wave 5 cross sectional)


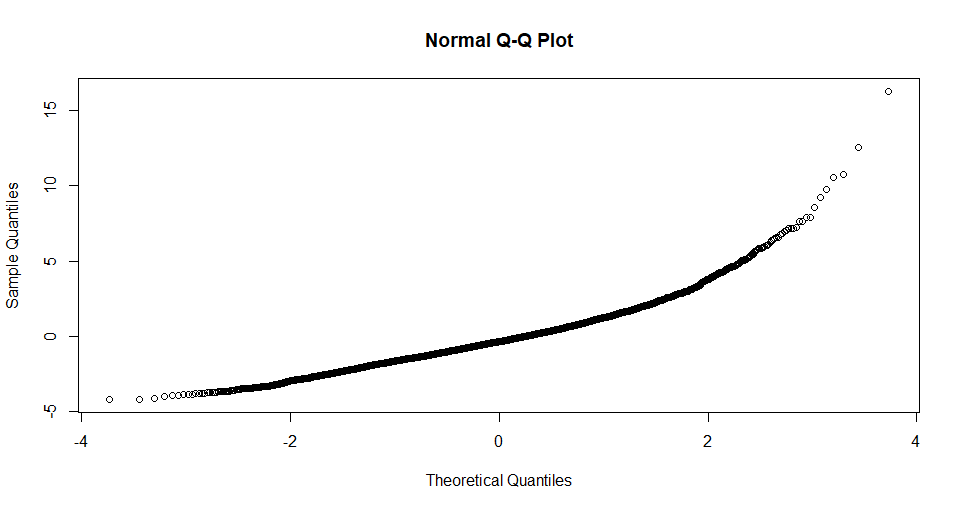
Visual inspection of Q-Q plot indicated non-normal distribution of residuals:

So, the outcome variable (network size was log transformed) in the following model:

Log (Network size) ~ age + sex + relationship + health + SES +Agreeableness + Extraversion + Verbal Communication, (weighting = wave 5 cross sectional)


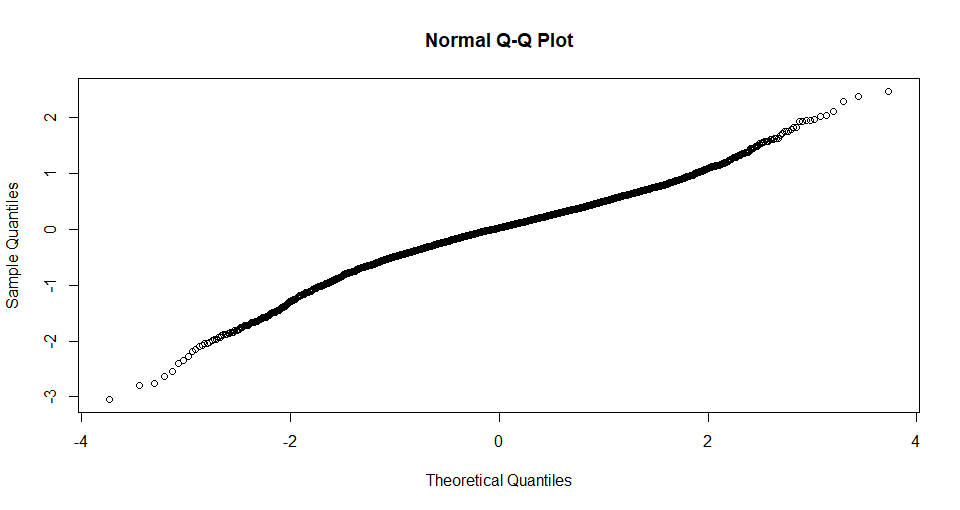
Visual inspection of Q-Q plot indicated approximately normal distribution of residuals:

Assessment of independence of errors and multicollinearity resulted in no violations, the data met the assumption of independent errors (Durbin-Watson value = 1.95; output = OK: Residuals appear to be independent and not autocorrelated (p = 0.146) and VIF values are presented below:

| Term | VIF | Increased SE | Tolerance |
| --- | --- | --- | --- |
| Age  Gender  Relationship status  Health  SES  Agreeableness  Extraversion  Verbal communication | 1.16  1.12  1.13  1.17  1.08  1.28  1.65  1.52 | 1.08  1.06  1.06  1.08  1.04  1.13  1.28  1.23 | 0.86  0.90  0.89  0.86  0.93  0.78  0.61  0.66 |

However, assumption checks indicated heteroscedasticity: output = Heteroscedasticity (non-constant error variance) detected (p < .001).

To counter heteroscedasticity a robust method was employed to calculate heteroscedasticity corrected standard errors. Using the ‘sandwich’ package in R, new coefficient standard errors were computed and used in the model tables.

***Inclusion of quadratic term for age***

Age^2^ was added to the full model and compared to the full model with no quadratic term for age. The model fit was improved, however there was the issue of multicollinearity shown through high VIF values for age and age^2^. Therefore, a mean-centered model was fit using: ‘jtools’ ‘center_mod’.

- - 1. **Friend Network Size**

Based on the following model:

Log (Friend network size) ~ age + sex + relationship + health + SES +Agreeableness + Extraversion + Verbal Communication


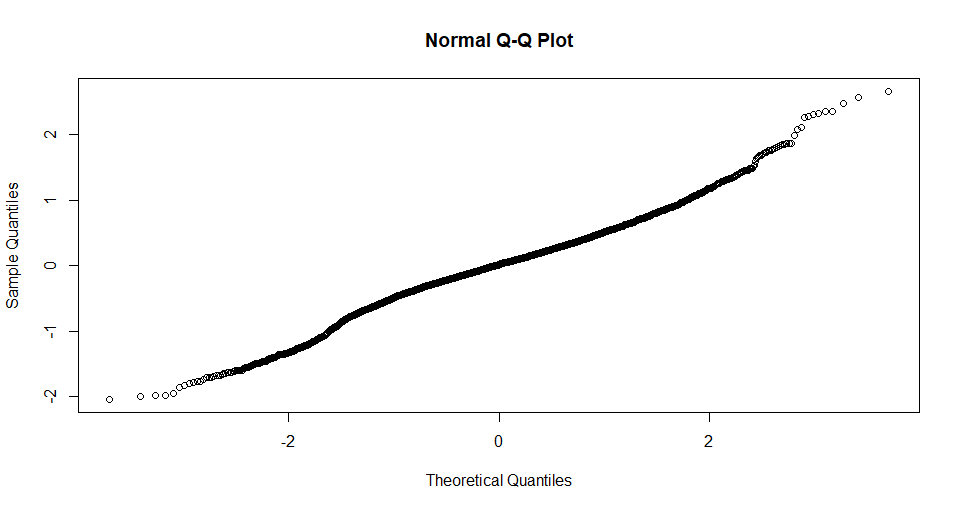
Visual inspection of Q-Q plot indicated an approximately normal distribution of residuals:

Assessment of heteroscedasticity (output = OK: Error variance appears to be homoscedastic (p = 0.738) and independence (output = OK: Residuals appear to be independent and not autocorrelated (p = 0.716) indicated no violations. Assessment of multicollinearity indicated no violations:

| Term | VIF | Increased SE | Tolerance |
| --- | --- | --- | --- |
| Age  Gender  Relationship status  Health  SES  Agreeableness  Extraversion  Verbal communication | 1.14  1.10  1.12  1.16  1.09  1.31  1.59  1.53 | 1.07  1.05  1.06  1.07  1.04  1.14  1.26  1.24 | 0.88  0.91  0.89  0.87  0.92  0.77  0.63  0.65 |

***Inclusion of quadratic term for age***

Age^2^ was added to the full model and compared to the full model with no quadratic term for age. The model fit was improved, however there was the issue of multicollinearity shown through high VIF values for age and age^2^. Therefore, a mean-centered model was fit using: ‘jtools’ ‘center_mod’.

- - 1. **Family Network Size**

Based on the following model:


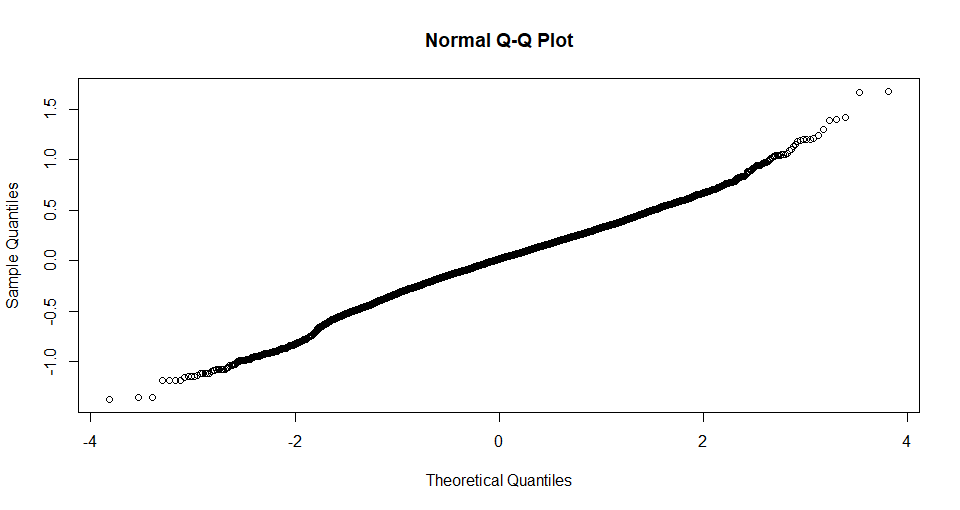
Log (Family network size) ~ age + sex + relationship + health + SES +Agreeableness + Extraversion + Verbal Communication

Visual inspection of Q-Q plot indicated an approximately normal distribution of residuals:

Assessment of independence (OK: Residuals appear to be independent and not autocorrelated (p = 0.888) and multicollinearity indicated no violations, see VIF values below.

| Term | VIF | Increased SE | Tolerance |
| --- | --- | --- | --- |
| Age  Gender  Relationship status  Health  SES  Agreeableness  Extraversion  Verbal communication | 1.14  1.11  1.12  1.20  1.07  1.29  1.72  1.55 | 1.07  1.05  1.06  1.10  1.03  1.14  1.31  1.25 | 0.88  0.90  0.89  0.83  0.94  0.77  0.58  0.64 |

However, assessment of heteroscedasticity indicated a violation of the assumption (output = Heteroscedasticity (non-constant error variance) detected (p < .001). To counter heteroscedasticity a robust method was employed to calculate heteroscedasticity corrected standard errors. Using the ‘sandwich’ package in R, new coefficient standard errors were computed and used in the model tables.

1. Additional Model Comparisons
   - 1. **Total Network Size**

Model comparison performed using the following command: Anova (model 1, model 2 test = "Chisq", method = "Wald")

**Model Comparison- one variable added to the null per model**

|  | df | R^2^ | Chi-squared against Null Model | AIC |
| --- | --- | --- | --- | --- |
| Null Model | 8 | .024 |  | 9309 |
| Null + VC | 9 | .043 | 80.67* | 9206 |
| Null + AG | 9 | .057 | 128.99* | 9131 |
| Null + EX | 9 | .067 | 184.40* | 9076 |
| Full Model | 11 | .078 | 229.93* | 9016 |

*Note.* Null Model included age, gender, relationship, health, socioeconomic status and controlled for household effect. Full model included VC, AG and EX. VC = verbal communication, AG = agreeableness, EX = extraversion.

**Model comparison stepwise for the variables of concern**

|  | Df | R^2^ | Chi-squared against model above | AIC |
| --- | --- | --- | --- | --- |
| Null Model | 8 | .024 |  | 9309 |
| Null + EX | 9 | .067 | 184.40* | 9076 |
| Null + EX + AG | 10 | .078 | 45.32* | 9015 |
| Full Model | 11 | .078 | 1.07(n.s.) | 9016 |
| Full Model + age^2^ | 12 | .080 | 7.75* | 9007 |

*Note.* Null Model included age, gender, relationship, health, socioeconomic status and controlled for household effect. Full model included VC, AG and EX. VC = verbal communication, AG = agreeableness, EX = extraversion.

1. Supplementary plots
   - 1. **Total Network Size**

*Figure. 3. Network Size by Extraversion and Agreeableness Scores (n=5202)*


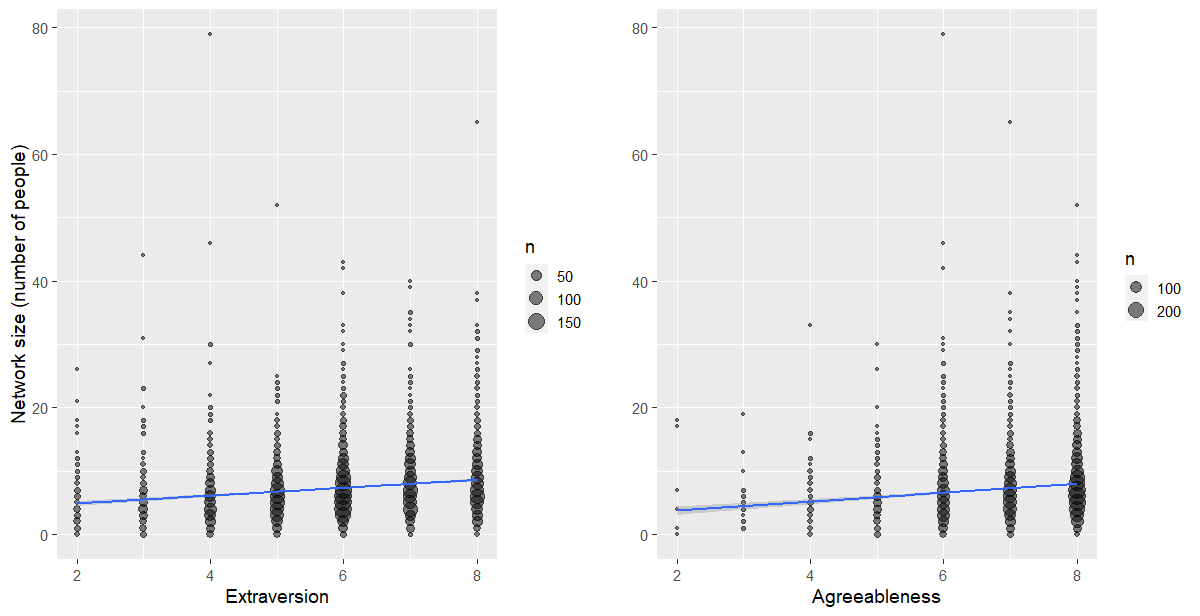


*Note.* Point size indicates the number of respondents, linear regression line and confidence intervals are presented.

1. Interpreting Regression Coefficients

In the final models the dependent variables are log-transformed, therefore a back-transformation has been applied to interpret the regression coefficients. Regression coefficients were interpreted using the following formula:

𝑒^𝛽̂1^ − 1

Which, for example, in the case of extraversion with the regression coefficient of 0.06 is:

(𝑒^0.06) -1 = 1.0618 -1 = 0.06

1. **Treating the predictor verbal communication as ordinal**

|  | Model with continuous predictor | | Model with ordinal predictor | |
| --- | --- | --- | --- | --- |
| *Predictor* | *B (CI)* | *p* | *B (CI)* | *p* |
| *Age*  *Age^2^*  *Sex*  *Relationship status*  *Health*  *SES8*  *Agreeableness*  *Extraversion*  *VC continuous*  *VC Linear*  *VC Quadratic*  *VC Cubic* | .003***  (.002, .004)  -.0003***  (-.0004, -.0002)  .100***  (.086, .114)  -.026***  (-.040, -.013)  .015***  (.008, .022)  .014***  (.010, .017)  .064***  (.055, .072)  .062***  (.055, .069)  .012**  (.002, .022) | <0.001  <0.001  <0.001  <0.01  <0.001  <0.001  <0.001  <0.001  <0.05 | .043***  (.031, .056)  -.0003***  (-.0004, -.0002)  .100***  (.086, .114)  -.026***  (-.039, -.012)  .015***  (.008, .022)  .013***  (.010, .017)  .064***  (.056, .073)  .062***  (.055, .068)  .060***  (.023, .098)  -.045***  (-.074, -.017)  .004  (-.013, .021) | <0.001  <0.001  <0.001  <0.01  <0.01  <0.001  <0.001  <0.001  <0.01  <0.01  .665 |
| Observations  Log Likelihood  AIC | 5,202  -4,493.716  9,007.432 | | 5,202  -4,491.223  9,006.447 | |

*Table. 3. Regression model results comparison for the treatment of verbal communication as continuous and ordinal*

*Note.* VC = Verbal Communication

Table 3 illustrates that by treating verbal communication as continuous or ordinal the model results do not significantly differ. For the model with the ordinal predictor the linear and quadratic polynomial contrasts fit the model better than the cubic polynomial contrast.

1. **Bootstrapping**

Using the R package ‘boot’ bias adjusted confidence intervals were calculated for the regression coefficients for the quadratic model (one of the final models in the cross-sectional analysis) and the total social network size change model (one of the final models in the change analysis). As can be seen in Table 4, the bias for each regression coefficient is very small, this also results in the bias-adjusted confidence intervals being very similar to those in the original regression.

*Table. 4. Linear regression model for the cross-sectional analysis with bootstrapping statistics and bias adjusted confidence intervals*

|  | **log(Network size with constant)** | | |  |  |  |
| --- | --- | --- | --- | --- | --- | --- |
| *Predictors* | *Original estimates* | *CI* | *bootBias* | *bootSE* | *BCa CI* |  |
| Age | 0.04 | 0.013 – 0.065 | 0.0001 | 0.014 | 0.011 - 0.066 |  |
| Age^2 | -0.00 | -0.0005 – -0.0001 | 0.000001 | 0.0001 | -0.0005 - 0.0001 |  |
| Gender | 0.09 | 0.062 – 0.127 | 0.0002 | 0.017 | 0.061 - 0.127 |  |
| Relationship status | -0.03 | -0.064 – 0.002 | 0.0004 | 0.017 | -0.064 - 0.002 |  |
| Health | 0.02 | 0.001 – 0.031 | 0.00002 | 0.008 | -0.0006 - 0.031 |  |
| SES | 0.01 | 0.004 – 0.020 | 0.00007 | 0.004 | 0.004 - 0.020 |  |
| Agreeableness | 0.06 | 0.044 – 0.077 | 0.000003 | 0.009 | 0.044 - 0.079 |  |
| Extraversion | 0.06 | 0.048 – 0.074 | 0.00004 | 0.007 | 0.046 - 0.074 |  |
| Verbal Communication | 0.02 | -0.006 – 0.037 | 0.0002 | 0.011 | -0.006 - 0.038 |  |
| Observations | 5202 | | |  |  |  |
| R^2^ / R^2^ adjusted | 0.077 / 0.075 | | |  |  |  |

*Note.* BCa = Bias Corrected. Number of Bootstrap Replications R=6000. This linear model does not include the sample weights used in the published analysis.

*Table. 5. Sample of 6 bootstrapped regression coefficients per variable of interest*

|  | Agreeableness | Extraversion | Verbal communication |
| --- | --- | --- | --- |
| 1 | 0.05033747 | 0.06241620 | 0.005744720 |
| 2 | 0.04825647 | 0.05871622 | 0.010895593 |
| 3 | 0.06397733 | 0.05146202 | 0.015019088 |
| 4 | 0.06637231 | 0.07018442 | 0.005581667 |
| 5 | 0.07003489 | 0.05313521 | 0.017878026 |
| 6 | 0.06061550 | 0.05654466 | 0.020912631 |

*Note.* Code to produce sample of bootstrapped coefficients: head(results$t, digits = 3)

As can be seen in Table 5, the bootstrapped regression coefficients, based on resampling of 6000 replications hover around similar coefficient values.

*Figure. 4. Histograms of bootstrap estimated regression coefficients for agreeableness, extraversion and verbal communication, the blue line represents the original regression coefficient from the linear model.*

*
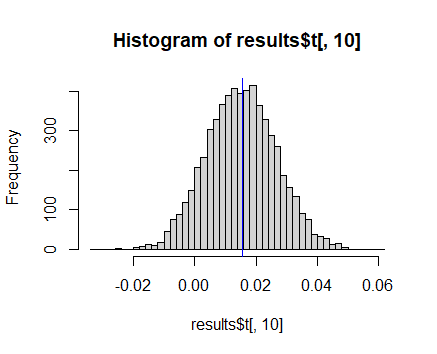

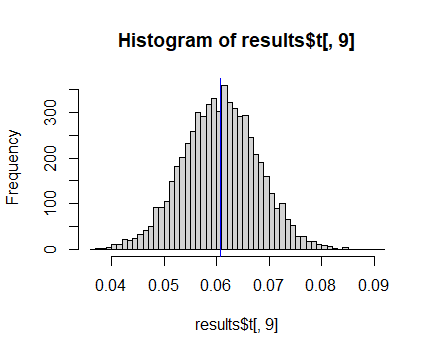

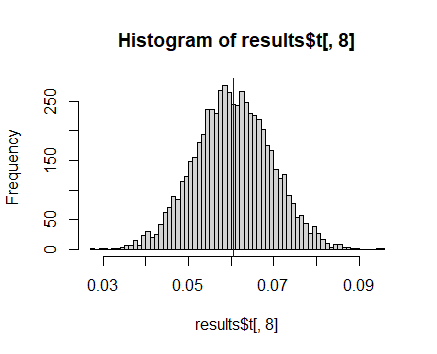
*As can be seen in Figure 4, the original regression coefficients for agreeableness, extraversion, and verbal communication, are aligned in the middle of the estimated coefficients based on the bootstrapping analysis with 6000 bootstrap replications.

*Table. 6. Regression model for the change analysis with bootstrapping statistics and bias adjusted confidence intervals*

|  | Social network size change | | | |
| --- | --- | --- | --- | --- |
| *Predictors* | *Original estimates* | *bootBias* | *BootSE* | *BCa CI* |
| Age at baseline | -0.01 | 0.0001 | 0.009 | -0.027 - 0.007 |
| Gender | -0.16 | -0.002 | 0.113 | -0.381 - 0.074 |
| Relationship at close | 0.06 | 0.0009 | 0.120 | -0.178 - 0.303 |
| General health at close | 0.07 | 0.0006 | 0.052 | -0.038 - 0.172 |
| Agreeableness | -0.08 | -0.001 | 0.055 | -0.191 - 0.031 |
| Extraversion | 0.08 | -0.00002 | 0.047 | -0.010 - 0.176 |
| Verbal communication | -0.06 | 0.0002 | 0.072 | -0.207 - 0.076 |
| Observations | 1511 | | | |

*Note.* BCa = Bias Corrected. Number of Bootstrap Replications R=4000. This model does not include the sample weights used in the published analysis.

*Table. 7. Sample of 6 bootstrapped regression coefficients per variable of interest*

|  | Agreeableness | Extraversion | Verbal communication |
| --- | --- | --- | --- |
| 1 | -0.106772449 | 0.078770137 | 0.018177939 |
| 2 | -0.035824520 | 0.046318716 | -0.001130897 |
| 3 | -0.006173344 | 0.037120482 | -0.049373073 |
| 4 | -0.154708065 | 0.100143469 | -0.087043113 |
| 5 | -0.079098349 | 0.003979374 | -0.033928997 |
| 6 | -0.100491088 | 0.075267534 | -0.061753609 |

*Note.* Code to produce sample of bootstrapped coefficients: head(results$t, digits = 3)

As can be seen in Table 7, the bootstrapped regression coefficients, based on resampling of 4000 replications, hover around similar coefficient values.

1. **References**

McCroskey, J. C., & McCroskey, L. L. (1988). Self‐report as an approach to measuring communication competence. *Communication Research Reports*, *5*(2), 108–113. https://doi.org/10.1080/08824098809359810

McCroskey, J. C., & Richmond, V. P. (1987). Willingness to communicate. In J. C. McCroskey & J. A. Daly (Eds.), *Personality and interpersonal communication* (pp. 119–131). Newbury Park, CA: Sage.

Rubin, R. B., & Martin, M. M. (1994). Development of a measure of interpersonal communication competence. *Communication Research Reports*, *11*(1), 33–44. https://doi.org/10.1080/08824099409359938

1. **R Packages**

R Studio Version 1.4.1717

| **Package** | **Version** | **Use** |
| --- | --- | --- |
| performance | 0.7.2 | Model diagnostics and comparison |
| survey | 4.1-1 | Applying survey weights |
| sandwich | 3.0-1 | Computing robust standard errors |
| lme4 | 1.1-27.1 | Models |
| stargazer | 5.2.2 | Tables |
| table1 | 1.4.2 | Tables |
| ggplot2 | 3.3.5 | Figures |
| boot | 1.3-28 | Bootstrapping |
